# Multi-modal characterization of the left atrium by a fully automated integration of pre-procedural cardiac imaging and electro-anatomical mapping

**DOI:** 10.1016/j.ijcha.2023.101276

**Published:** 2023-10-11

**Authors:** Ben J.M. Hermans, Geertruida P. Bijvoet, Robert J. Holtackers, Casper Mihl, Justin G.L.M. Luermans, Bart Maesen, Kevin Vernooy, Dominik Linz, Sevasti-Maria Chaldoupi, Ulrich Schotten

**Affiliations:** a Department of Physiology, Cardiovascular Research Institute Maastricht (CARIM), Maastricht University, Maastricht, the Netherlands; b Department of Cardiology, Cardiovascular Research Institute Maastricht (CARIM), Maastricht University Medical Center (MUMC+), Maastricht, the Netherlands; c Department of Radiology and Nuclear Medicine, Cardiovascular Research Institute Maastricht (CARIM), Maastricht University Medical Center (MUMC+), Maastricht, the Netherlands; d Department of Cardiothoracic Surgery, Cardiovascular Research Institute Maastricht (CARIM), Maastricht University Medical Center (MUMC+), Maastricht, the Netherlands

**Keywords:** Atrial fibrillation, Imaging, Mapping, Alignment, Integration, clinicaltrials.gov NCT04342312, Netherlands Trial Register NL7894

## Abstract

**Background:**

The combination of information obtained from pre-procedural cardiac imaging and electro-anatomical mapping (EAM) can potentially help to locate new ablation targets. In this study we developed and evaluated a fully automated technique to align left atrial (LA) anatomies obtained from CT- and MRI-scans with LA anatomies obtained from EAM.

**Methods:**

Twenty-one patients scheduled for a pulmonary vein (PV) isolation with a pre-procedural MRI were enrolled. Additionally, a recent computed tomography (CT) scan was available in 12 patients. LA anatomies were segmented from MRI-scans using ADAS-AF (Galgo Medical, Barcelona) and from the CT-scans using Slicer3D. MRI and CT anatomies were aligned with the EAM anatomy using an iterative closest plane-to-plane algorithm. Initially, the algorithm included the PVs, LA appendage and mitral valve anulus as they are the most distinctive landmarks. Subsequently, the algorithm was applied again, excluding these structures, with only three iterative steps to refine the alignment of the true LA surface. The result of the alignments was quantified by the Euclidian distance between the aligned anatomies after excluding PVs, LA appendage and mitral anulus.

**Results:**

Our algorithm successfully aligned 20/21 MRI anatomies and 11/12 CT anatomies with the corresponding EAM anatomies. The average median residual distances were 1.9 ± 0.6 mm and 2.5 ± 0.8 mm for MRI and CT anatomies respectively. The average LA surface with a residual distance less than 5.00 mm was 89 ± 9% and 89 ± 10% for MRI and CT anatomies respectively.

**Conclusion:**

An iterative closest plane-to-plane algorithm is a reliable method to automatically align pre-procedural cardiac images with anatomies acquired during ablation procedures.

## 1 Introduction

Pulmonary vein isolation (PVI) represents the cornerstone of modern atrial fibrillation (AF) rhythm control strategies [1], [2]. However, despite novel ablation strategies and techniques to localize and target extra-pulmonary vein drivers,[3], [4], [5], [6] the success rates in patients with persistent AF remains suboptimal [7]. Currently proposed techniques either seek to detect ablation targets by the identification of electrical properties of the atria (bipolar voltages or specific propagation pattern) through electro-anatomical mapping (EAM) or by the identification of structural remodeling by pre-procedural cardiac imaging. For example, STAR AF II showed that additional ablations guided by electrophysiological properties do not improve outcome compared to PVI-only [7]. On the other hand, although the degree of atrial fibrosis identified by late gadolinium enhancement (LE) magnetic resonance imaging (MRI) is associated with post-PVI AF recurrence [8], an MRI-guided ablation did not outperform a routine PVI [9]. A multi-modal characterization of the left atrium (LA) that integrates (structural) data obtained from pre-procedural images with (electrophysiological) EAM data obtained during the procedure can bring new possibilities to the field of AF ablations. Moreover, the emerging area of personalized modeling for the identification of ablation targets could introduce novel instruments to the field of electrophysiology procedures [10], [11], [12]. However, a requirement of these integrated multi-modal targeted AF ablation approach would be a reliable alignment between left atrial (LA) anatomies derived from pre-procedural cardiac imaging and EAM. Existing EAM systems align segmented pre-procedural images and EAM anatomies through either entirely visual manual alignment (i.e. manually dragging and rotating the pre-procedural anatomy until the user is satisfied with the alignment) or by an alignment based on manually chosen landmarks. In this paper we present and evaluate a fully automatic technique to align LA anatomies derived from pre-procedural CT- and MRI-scans with LA anatomies derived from EAM.

## 2 Materials & methods

### 2.1 Study population

This study enrolled 21 patients scheduled for a redo pulmonary vein (PV) isolation procedure. Prior to the ablation, all patients underwent a 3D LGE MRI scan. Additionally, computed tomography (CT) scans performed within 5 years prior to the ablation were exported for analysis.

Patients enrolled in this study were included in the ISOLATION registry [13] which was reviewed and approved by the ethics committee of the Maastricht University Medical Centre (METC azM/UM, NL70787.068.19) and is registered at clinicaltrials.gov (NCT04342312) and Netherlands Trial Register (NL7894).

### 2.2 Cardiac imaging acquisition

The LGE MRI was performed using a 1.5 T clinical MR system (Ingenia, Philips Healthcare, Best, the Netherlands). An ECG-triggered respiratory-navigated high-resolution 3D whole heart dark-blood LGE scan was performed 10 min after an intravenous injection of 0.2 mmol/kg gadobutrol (Gadovist; Bayer Pharmaceuticals, Berlin, Germany) with a steadily increasing inversion time as described earlier [14], [15]. The obtained in-plane reconstructed resolution was 0.625 × 0.625 × 1 mm (acquired 1.25 x1.25 × 2 mm).

Cardiac CT imaging was conducted using a third-generation dual source CT-scanner (Somatom Force, Siemens Healthineers, Forchheim, Germany; slice collimation 192x0.6 mm). Tube voltage of scan protocols varied between 70 and 100 kV. Slice thickness was 0.6 mm. The acquisition window was set to acquire mid-diastolic cardiac phases at 65% of the R-R interval. Contrast injection consisted of 300 mg l/ml Iopromide (Ultravist, Bayer Healthcare, Berlin, Germany), injected using a dual-head CT power injector (Stellant, Bayer). After a test bolus of 15 ml with saline flush, a body weight adapted contrast medium injection protocol was used (resulting in an average total bolus of 40–60 ml) followed by another saline flush. By default, a prospectively ECG-triggered ‘high pitch’ spiral protocol was used. A retrospectively gated helical protocol was used in one case of an irregular heart rhythm and heart rate ≥ 90 bpm.

### 2.3 Cardiac imaging segmentations

An experienced imaging-cardiologist (GPB), who was blinded to the EAM anatomy, manually segmented the left atrial (LA) wall in both the LGE MRI and CT scan images using ADAS-AF (Galgo Medical SL, Barcelona, Spain) and Slicer3D (slicer.org [16]), respectively. To assess the intra- and interobserver variability of the LGE MRI segmentations, three MRI scans were segmented twice by the first observer (GB) and once by a second observer (SMC). In regard to the segmentation of CT-scans, no intra- or interobserver variability was assessed as this segmentation employed ‘growing seed points’ based on Hounsfield units, which reduced susceptibility to varying interpretations. The segmented LA anatomies were exported as text files for further analysis.

### 2.4 Electro-anatomical mapping

All patients underwent a catheter ablation under general anesthesia or deep sedation. A right femoral access was used to advance the catheters. Transseptal puncture was performed guided by transesophageal ultrasound imaging. Prior to any ablations a high-density LA EAM was recorded during sinus rhythm using the Advisor™ HD Grid Mapping Catheter with the EnSite Velocity system (Abbott, St. Paul, Minnesota, USA) or the Pentaray catheter with the CARTO system (Biosense Webster, Diamond Bar, CA, USA). Field scaling was enabled during all EnSite procedures. Patients were treated and observed for 24 h according to routine clinical practice. EAM anatomies were exported for offline alignment and analysis.

### 2.5 Alignment

LA anatomies obtained from MRI/CT and EAM were aligned using an iterative closest plane-to-plane (ICP-PP) algorithm in MATLAB 2022b (*pcregistericp*, Computer Vision Toolbox, Mathworks, Natick, MA, USA). ICP-PP is a variant of the Iterative Closest Point (ICP) algorithm, which is a widely used technique for aligning two sets of point clouds in 3D space. In ICP-PP, the two point clouds are assumed to be sampled points of planar surfaces, rather than distinct points. The algorithm seeks to find the best transformation (translation and rotation) that aligns the planes of the two point clouds whereas a ICP algorithm seeks to find the best alignment between distinct points.

To align the MRI and CT LA anatomies with the EAM, the ICP-PP algorithm was first applied on the entire segmentations including the PVs and mitral valve anulus since the PVs are the most distinct landmarks in the LA anatomies. To further refine the alignment, the ICP-PP algorithm was applied once more after excluding the PVs and mitral valve anulus. In this final refinement step, only 3 iterative steps were allowed to ensure that the algorithm only refines the previous alignment.

The MRI segmentations exported from ADAS-AF were found to have a non-isotropic mesh, meaning that the distance between 3D points is not equal. As a result, certain regions of the anatomy have far more points than others. To ensure accurate alignment, all MRI segmentations exported from ADAS-AF were remeshed to an isotropic mesh with equal distances between points before alignment.

After the alignment, a visual check was conducted by a single observer to confirm the correctness of the alignment. For the alignments that were deemed correct, the quality of alignment was quantified by calculating the Euclidean distance between all points of the aligned anatomies after exclusion of LA appendage, PVs and mitral valve annulus. The Euclidean distances were visualized on the MRI or CT anatomy to see regional distances. To further assess the alignment quality, the percentage of the LA that fell below 2.5 mm and 5 mm were calculated.

## 3 Results

### 3.1 Study population

A total of 21 patients were enrolled. As summarized in Table 1, the study population was: 62% male; age, 65 ± 8 years; BMI, 28 ± 4 kg/m^2^; left atrium volume index, 43 ± 11 ml/m^2^. A CT-scan within 5 years of the ablation procedure was available for 12 of the 21 enrolled patients. The median interval between MRI and the ablation procedure was 96 days (range: 21–307). The interval between the available CT-scans and the ablation procedure was longer with a median interval of 972 days (range: 161–1785). The median number of vertices in MRI, CT and EAM anatomies were 22,494 (range: 14,414–35,130), 145,128 (range: 116,442–208,482), and 14,323.5 (range: 8,557–80,256) respectively.

**Table 1:** Study population characteristics.

	MRI (n = 21)		CT (n = 12)
Age (years)	65 ± 8		66 ± 7
Men	13 (62%)		6 (50%)
Interval CT/MRI & EAM (days)	96 [21–307]		972 [161–1785]
Carto	14 (62%)		7 (58%)
Ensite	8 (38%)		5 (42%)
BMI (kg/m^2^)	28 ± 4		28 ± 5
BSA (m^2^)	2.0 ± 0.3		2.0 ± 0.3
LAVI (ml/m^2^)	43 ± 11		43 ± 13

Data represented as mean ± standard deviation, count (percentage%) or median [min – max]. CT, computed tomography; MRI, magnetic resonance imaging; EAM, electro-anatomical mapping; BMI, body mass index; BSA, body surface area; LAVI, left atrial volume index.

### 3.2 Alignment results

**Fig. 1:**
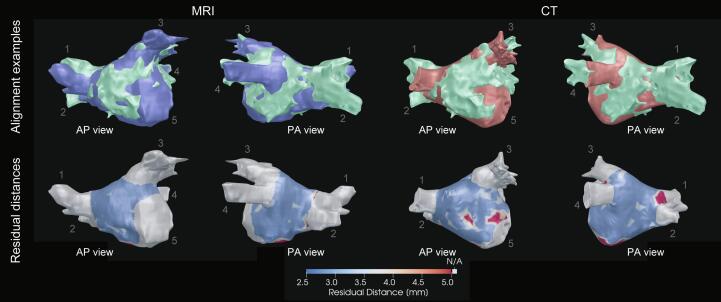
**Alignment examples** The top row shows anterior-poster (AP) and posterior-anterior (PA) views of alignment examples of one patient’s MRI (blue, left) and CT anatomy (right, red) with the corresponding EAM anatomy (green). The bottom row shows the regional residual Euclidean distances between the aligned anatomies (bottom). 1, Right superior PV; 2, Right inferior PV; 3, Left superior PV; 4, Left inferior PV; 5, Mitral valve anulus; MRI, magnetic resonance imaging; CT, computed tomography; EAM, electro-anatomical mapping. (For interpretation of the references to colour in this figure legend, the reader is referred to the web version of this article.)

**Fig. 2:**
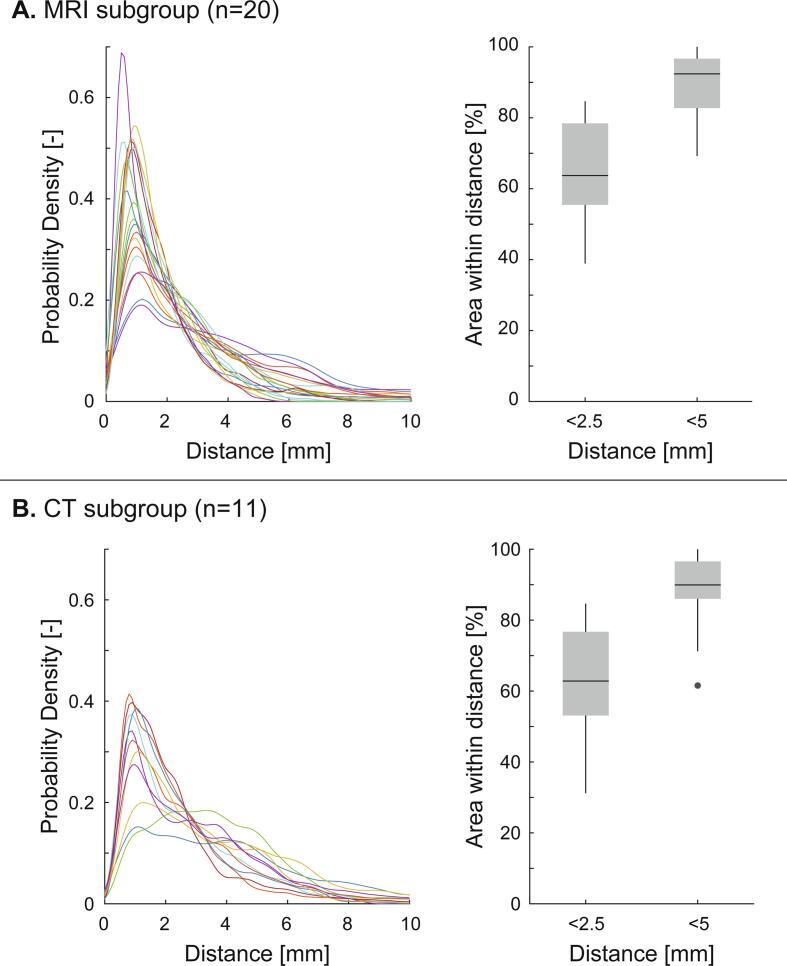
**Residual distances.** Panel A shows the results for the alignment of MRI scans, panel B the alignment of CT scans. The distribution of the residual Euclidean distances is shown on the left with colors indicating individual patients. The percentage of left atrial surface with a residual distance less than 2.5 mm and 5 mm are shown on the right. MRI, magnetic resonance imaging; CT, computed tomography.

**Fig. 3:**
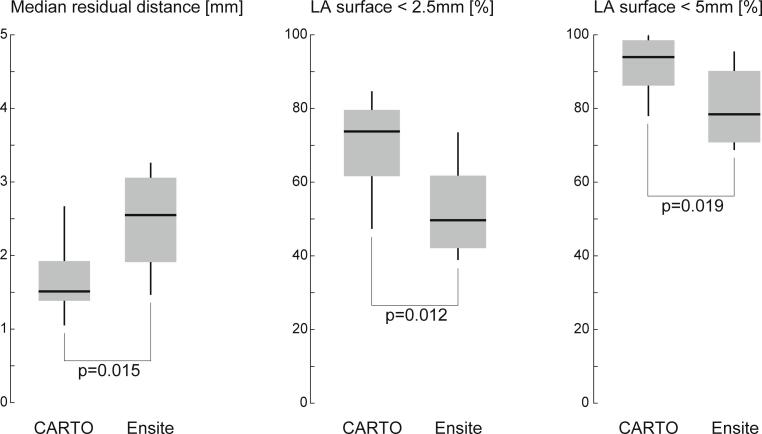
**CARTO resulted in a significant better alignment.** CARTO anatomies exhibited significantly smaller median residual distances (1.5 [1.4–1.9] mm compared to 2.6 [1.9 – 3.1] mm for Ensite, p = 0.015), a larger area with distances less than 2.5 mm (74 [62 – 80]% for CARTO versus 50 [42 – 62]% for Ensite, p = 0.012), and a larger area with distances less than 5.0 mm (94 [86 – 99]% for CARTO versus 80 [71 – 90]% for Ensite, p = 0.019). This analysis was carried out using a Wilcoxon rank sum test.

After exclusion of the LA appendage, PVs and MV annulus, our algorithm successfully aligned 20/21 MRI anatomies and 11/12 CT anatomies with the corresponding EAM anatomies based on visual inspection. Fig. 1 contains two examples of the alignment of the LA anatomy (top row) derived from an MRI scan (left) and CT scan (right) with the EAM LA anatomy for one patient. The bottom row shows the regional residual distance after alignment. The average median residual distance was 1.9 ± 0.6 mm for MRI anatomies and 2.5 ± 0.8 mm for CT anatomies respectively. The distributions of residual distances shown in Fig. 2 show that after alignment most of the LA is within 5.0 mm for both the MRI and CT anatomies. For the MRI anatomies (Fig. 2**A**), on average 89 ± 9% of the LA surface has a residual distance less than 5.0 mm and 65 ± 14% of the LA surface has a residual distance less than 2.5 mm. For the CT anatomies (Fig. 2**B**), on average 89 ± 10% of the LA surface has a residual distance less than 5.0 mm and 62 ± 16% of the LA surface has a residual distance less than 2.5 mm. CARTO EAM anatomies had a significant better alignment with MRI anatomies compared to Ensite EAM anatomies based on residual median distances and LA surface with a residual distance less than 2.5 mm and 5 mm (See Fig. 3).

**Fig. 4:**
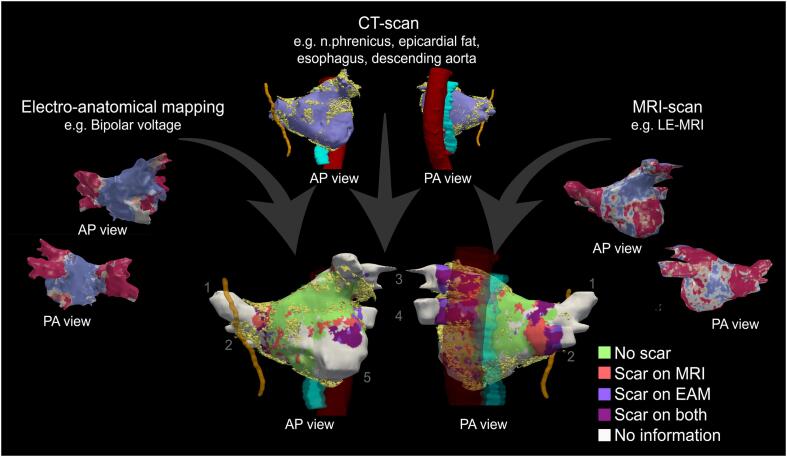
**Example of a multi-modal re-do PVI roadmap.** Alignment of different modalities allow for a multi-modal assessment of the left atrium and its relation to anatomical structures in its vicinity. In this example ‘scar’ was determined by low bipolar voltage in EAM and high signal intensity on LE-MRI. 1, Right superior PV; 2, Right inferior PV; 3, Left superior PV; 4, Left inferior PV; 5, Mitral valve anulus; AP view, anterior-posterior view; PA view, posterior-anterior view; CT, computed tomography; MRI, magnetic resonance imaging; LE, late gadolinium enhancement; EAM, electro-anatomical mapping.

An example of an automatic alignment of both CT and LA anatomies from CT, MRI and EAM is shown in Fig. 4. This alignment enables a multi-modal assessment of the left atrium and its relation to anatomical structures in its vicinity and can therefore serve as a roadmap for re-do procedures.

The intra- and interobserver variability of MRI-scan segmentations was low for all three MRI-scans with an average median distance of 1.0 mm and 1.3 mm respectively (See Supplementary Table S1**)**.

## 4 Discussion

In this paper, we present a novel technique for automatic alignment of segmented preprocedural LA MRI and CT images with procedural EAM. The interest in using preprocedural cardiac images during AF ablation procedures has diminished as the quality of the anatomy derived during catheter-based EAM procedures has improved significantly over the past decade. However, recent advances in cardiac imaging providing information on extracellular matrix formation, ablation lesion assessment and extra-cardiac structures have renewed interest in aligning preprocedural images [17], [18].

### 4.1 Future perspectives and clinical implementation

The herein presented alignment strategy for the integration of preprocedural imaging with anatomies from EAM allows integrated multi-modal assessment of the LA. Although previous attempts to perform ablations guided by preprocedural images were not successful, advancements in the field of cardiac imaging might change this. For example, a recent study showed that the novel dark-blood LE-MRI scans improved atrial scar evaluation compared to the conventional bright-blood LE-MRI because it improves the contrast between scar and blood in LE-MRI scans [19]. Another recent study performed a personalized PVI guided by left atrial wall thickness which could lead to better transmural and more durable lesions [20]. These innovations can only lead to image-guided ablations if the preprocedural images can successfully be aligned with the anatomical data obtained during the EAM of the procedure.

Furthermore, the translation and rotation used to align the LA CT anatomy with the EAM anatomy can also be applied to other anatomical structures that can only be seen on the CT-scan, such as epicardial fat, the descending aorta, the esophagus, and the phrenic nerve as shown in Fig. 4. By combining these features obtained from CT scans with LGE MRI and bipolar voltage, we can create a roadmap that can be used to perform a redo PVI, providing a holistic and multi-modal assessment of the AF substrate. Additionally, this roadmap enhances procedural safety by providing a clear visualization of anatomical structures that cannot be visualized with EAM alone (e.g., the phrenic nerve and esophagus). Overall, such alignments may enable more accurate and safe procedures for the treatment of AF but also enables multi-modal studying the arrhythmia substrate in research.

### 4.2 Other integration algorithms

Both the CARTO and EnSite systems have built-in image registration tools called CartoMerge [21] and NavX fusion [22] respectively. A randomized comparison between the two showed that the CartoMerge algorithm was faster and used less fluoroscopy while overall procedural times and clinical outcomes were similar between the two algorithms [23]. The advantages of these algorithms is that they enable a live import and integration of previously made cardiac images during ablation procedures. A drawback of these algorithms is their dependence on the selection of only a few anatomical landmarks to align the anatomies of CT/MRI and EAM. Our iterative closest plane-to-plane algorithm takes all points of the anatomies into account and does not require any manual landmarks. This does not only make the algorithm fully automated, it furthermore utilizes all available information of the anatomies and thereby reduces the ambiguity of an alignment.

Other non-commercial algorithms have been described but are often more than 10 years old and therefore still had to deal with the low-resolution EAM anatomies of those days [24], [25], [26]. Shu et al. more recently published another automatic approach to align preprocedural images with EAM but only assessed its performance in 6 patients [27]. Roney et al. recently introduced a new method for multi-modal assessment, which involves transforming 3D LA anatomy into a 2D representation called universal atrial coordinates [28]. This enables an easy comparison of LA surfaces between modalities but also across patients and therefore has great potential for future studies that for example attempt to unravel the interplay between functional and structural substrates of AF. However, a drawback of this approach is that the 3D information is lost and the LA appendage is flattened, which limits its direct use in ablation procedures. We introduce and assess an approach, which can be used to align several imaging modalities (CT and MRI) with EAM, while preserving the true 3D information, which could be integrated in future ablation strategies guided by data derived by combined multi-modal information.

### 4.3 Limitations

Our algorithm cannot account for differences in LA volume between the cardiac images and the ablation procedure. For instance, if there is a change in end-diastolic volume (e.g. because of changes in volume status), the alignment may not be accurate. The differences in anatomies caused by a different end-diastolic volume will remain after the alignment. To minimize the residual error resulting from anatomical discrepancies, it is crucial to minimize the time gap between the MRI/CT scan and the EAM procedure, and furthermore ensure that both sets of anatomical data are acquired during the same phase of the cardiac cycle. Some previous algorithms, such as NavX fusion [22], have addressed this issue by allowing for differences in volume and shape in the segmentations. However, we decided not to include regional scaling or morphing, as it could introduce additional uncertainties in the alignment process. It is likely that not all regions of the LA are equally affected by a different volume status. For example, the posterior wall most likely is less suspectable to volume changes because of the fixed position of the PVs. We believe that it is preferable to rather exclude unreliable alignments than to attempt to force an alignment by scaling or morphing the anatomies. This is because even after scaling or morphing, the alignment of anatomies with differences in volume status is very questionable.

Since our algorithm is not implemented in the navigation systems, it can only be used offline after exporting the EAM anatomies. Therefore, at this moment, the roadmap as shown in Fig. 4 cannot be generated during the procedure.

Unfortunately, a recent CT-scan was not available from all patients. The interval between the available CT-scans and the ablation procedure was longer, with a median duration of 972 days, compared to the median duration of 96 days between the MRI-scans and the ablation procedure. This prolonged interval most likely lead to the somewhat increased residual distances between CT and EAM anatomies compared to the residual distances between MRI and EAM anatomies.

## 5 Conclusion

In conclusion, our study demonstrates that an iterative closest plane-to-plane algorithm is a reliable method to align pre-procedural with anatomies acquired during ablation procedures. This approach allows for a comprehensive, multi-modal assessment of the left atrium. Whether this novel alignment approach can be integrated in future rhythm control strategies to guide and improve AF ablation warrants further study.

## Funding

This work was supported by the Netherlands Heart Foundation (CVON2014-09, RACE V Reappraisal of Atrial Fibrillation: Interaction between hyperCoagulability, Electrical remodeling, and Vascular Destabilisation in the Progression of AF, and Grant number 01-002-2022-0118, EmbRACE: Electro-Molecular Basis and the theRapeutic management of Atrial Cardiomyopathy, fibrillation and associated outcomEs), the European Union (ITN Network Personalize AF: Personalized Therapies for Atrial Fibrillation: a translational network, grant number 860974; MAESTRIA: Machine Learning Artificial Intelligence Early Detection Stroke Atrial Fibrillation, grant number 965286).

## CRediT authorship contribution statement

**Ben J.M. Hermans:** Conceptualization, Methodology, Software, Formal analysis, Writing – original draft, Visualization. **Geertruida P. Bijvoet:** Validation, Data curation, Writing – original draft. **Robert J. Holtackers:** Resources, Writing – review & editing. **Casper Mihl:** Resources, Writing – review & editing. **Justin G.L.M. Luermans:** Investigation, Writing – review & editing. **Bart Maesen:** Investigation, Writing – review & editing. **Kevin Vernooy:** Investigation, Writing – review & editing. **Dominik Linz:** Investigation, Supervision, Writing – review & editing. **Sevasti-Maria Chaldoupi:** Investigation, Validation, Data curation, Supervision, Writing – review & editing. **Ulrich Schotten:** Resources, Conceptualization, Supervision, Writing – review & editing.

## Declaration of Competing Interest

The authors declare that they have no known competing financial interests or personal relationships that could have appeared to influence the work reported in this paper.

## References

[1] Packer D.L., Mark D.B., Robb R.A., Monahan K.H., Bahnson T.D., Poole J.E., Noseworthy P.A., Rosenberg Y.D., Jeffries N., Mitchell L.B., Flaker G.C., Pokushalov E., Romanov A., Bunch T.J., Noelker G., Ardashev A., Revishvili A., Wilber D.J., Cappato R., Kuck K.-H., Hindricks G., Davies D.W., Kowey P.R., Naccarelli G.V., Reiffel J.A., Piccini J.P., Silverstein A.P., Al-Khalidi H.R., Lee K.L. (2019). for the CABANA Investigators, Effect of Catheter Ablation vs Antiarrhythmic Drug Therapy on Mortality, Stroke, Bleeding, and Cardiac Arrest Among Patients With Atrial Fibrillation: The CABANA Randomized Clinical Trial. J. Am. Med. Assoc..

[2] Inoue K., Hikoso S., Masuda M., Furukawa Y., Hirata A., Egami Y., Watanabe T., Minamiguchi H., Miyoshi M., Tanaka N., Oka T., Okada M., Kanda T., Matsuda Y., Kawasaki M., Hayashi K., Kitamura T., Dohi T., Sunaga A., Mizuno H., Nakatani D., Sakata Y., the OCVC Arrhythmia Investigators (2021). Pulmonary vein isolation alone vs. more extensive ablation with defragmentation and linear ablation of persistent atrial fibrillation: the EARNEST-PVI trial. EP Eur..

[3] Nademanee K., McKenzie J., Kosar E., Schwab M., Sunsaneewitayakul B., Vasavakul T., Khunnawat C., Ngarmukos T. (2004). A new approach for catheter ablation of atrial fibrillation: mapping of the electrophysiologic substrate. J. Am. Coll. Cardiol..

[4] Narayan S.M., Krummen D.E., Shivkumar K., Clopton P., Rappel W.-J., Miller J.M. (2012). Treatment of atrial fibrillation by the ablation of localized sources: CONFIRM (Conventional Ablation for Atrial Fibrillation With or Without Focal Impulse and Rotor Modulation) trial. J. Am. Coll. Cardiol..

[5] Seitz J., Bars C., Théodore G., Beurtheret S., Lellouche N., Bremondy M., Ferracci A., Faure J., Penaranda G., Yamazaki M., Avula U.M.R., Curel L., Siame S., Berenfeld O., Pisapia A., Kalifa J. (2017). AF Ablation Guided by Spatiotemporal Electrogram Dispersion Without Pulmonary Vein Isolation: A Wholly Patient-Tailored Approach. J. Am. Coll. Cardiol..

[6] Berenfeld O., Mandapati R., Dixit S., Skanes A.C., Chen J., Mansour M., Jalife J. (2000). Spatially distributed dominant excitation frequencies reveal hidden organization in atrial fibrillation in the Langendorff-perfused sheep heart. J. Cardiovasc. Electrophysiol..

[7] Verma A., Jiang C., Betts T.R., Chen J., Deisenhofer I., Mantovan R., Macle L., Morillo C.A., Haverkamp W., Weerasooriya R., Albenque J.-P., Nardi S., Menardi E., Novak P., Sanders P. (2015). Approaches to Catheter Ablation for Persistent Atrial Fibrillation. N. Engl. J. Med..

[8] Marrouche N.F., Wilber D., Hindricks G., Jais P., Akoum N., Marchlinski F., Kholmovski E., Burgon N., Hu N., Mont L., Deneke T., Duytschaever M., Neumann T., Mansour M., Mahnkopf C., Herweg B., Daoud E., Wissner E., Bansmann P., Brachmann J. (2014). Association of Atrial Tissue Fibrosis Identified by Delayed Enhancement MRI and Atrial Fibrillation Catheter Ablation: The DECAAF Study. J. Am. Med. Assoc..

[9] Marrouche N.F., Wazni O., McGann C., Greene T., Dean J.M., Dagher L., Kholmovski E., Mansour M., Marchlinski F., Wilber D., Hindricks G., Mahnkopf C., Wells D., Jais P., Sanders P., Brachmann J., Bax J.J., Morrison-de Boer L., Deneke T., Calkins H., Sohns C., Akoum N., DECAAF II Investigators, Abdul Karim A., Costea A., Leber A., Lubinski A., Elvan A., Herweg B., Koplan B., Jones C., Mahnkopf C., Sohns C., Wells D., Wilber D., Packer D., Daoud E., Atienza F., Bisbal F., Marchlinski F., Hindricks G., Pontone G., Estner H., Puererfellner H., Ramanna H., Calkins H., Brachmann J., Kalman J., Siebels J., Boersma L., Gotte M., Vloka M., Duytschaever M., Lluís M., Mansour M., Marrouche N., Akoum N., Wazni O., Kistler P., Jais P., Sanders P., Wakili R., Weerasooriya R., Nazarian S., Gautam S., Mittal S., Deneke T., Maurer T., Neumann T., Reddy V., Spear W. (2022). Effect of MRI-Guided Fibrosis Ablation vs Conventional Catheter Ablation on Atrial Arrhythmia Recurrence in Patients With Persistent Atrial Fibrillation: The DECAAF II Randomized Clinical Trial. J. Am. Med. Assoc..

[10] Boyle P.M., Zghaib T., Zahid S., Ali R.L., Deng D., Franceschi W.H., Hakim J.B., Murphy M.J., Prakosa A., Zimmerman S.L., Ashikaga H., Marine J.E., Kolandaivelu A., Nazarian S., Spragg D.D., Calkins H., Trayanova N.A. (2019). Computationally guided personalized targeted ablation of persistent atrial fibrillation. Nat. Biomed. Eng..

[11] Roney C.H., Beach M.L., Mehta A.M., Sim I., Corrado C., Bendikas R., Solis-Lemus J.A., Razeghi O., Whitaker J., O’Neill L., Plank G., Vigmond E., Williams S.E., O’Neill M.D., Niederer S.A. (2020). In silico Comparison of Left Atrial Ablation Techniques That Target the Anatomical, Structural, and Electrical Substrates of Atrial Fibrillation. Front. Physiol..

[12] Roy A., Varela M., Chubb H., MacLeod R., Hancox J.C., Schaeffter T., Aslanidi O. (2020). Identifying locations of re-entrant drivers from patient-specific distribution of fibrosis in the left atrium. PLoS Comput. Biol..

[13] Verhaert D.V.M., Linz D., Chaldoupi S.M., Westra S.W., den Uijl D.W., Philippens S., Kerperien M., Habibi Z., Vorstermans B., ter Bekke R.M.A., Beukema R.J., Evertz R., Hemels M.E.W., Luermans J.G.L.M., Manusama R., Lankveld T.A.R., van der Heijden C.A.J., Bidar E., Hermans B.J.M., Zeemering S., Bijvoet G.P., Habets J., Holtackers R.J., Mihl C., Nijveldt R., van Empel V.P.M., Knackstedt C., Simons S.O., Buhre W.F.F.A., Tijssen J.G.P., Isaacs A., Crijns H.J.G.M., Maesen B., Vernooy K., Schotten U. (2022). Rationale and Design of the ISOLATION Study: A Multicenter Prospective Cohort Study Identifying Predictors for Successful Atrial Fibrillation Ablation in an Integrated Clinical Care and Research Pathway. Front. Cardiovasc. Med..

[14] Holtackers R.J., Chiribiri A., Schneider T., Higgins D.M., Botnar R.M. (2017). Dark-blood late gadolinium enhancement without additional magnetization preparation. J. Cardiovasc. Magn. Reson..

[15] Holtackers R.J., Gommers S., Van De Heyning C.M., Mihl C., Smink J., Higgins D.M., Wildberger J.E., ter Bekke R.M.A. (2021). Steadily Increasing Inversion Time Improves Blood Suppression for Free-Breathing 3D Late Gadolinium Enhancement MRI With Optimized Dark-Blood Contrast. Invest. Radiol..

[16] Fedorov A., Beichel R., Kalpathy-Cramer J., Finet J., Fillion-Robin J.-C., Pujol S., Bauer C., Jennings D., Fennessy F., Sonka M., Buatti J., Aylward S., Miller J.V., Pieper S., Kikinis R. (2012). 3D Slicer as an image computing platform for the Quantitative Imaging Network. Magn. Reson. Imaging.

[17] Zghaib T., Keramati A., Chrispin J., Huang D., Balouch M.A., Ciuffo L., Berger R.D., Marine J.E., Ashikaga H., Calkins H., Nazarian S., Spragg D.D. (2018). Multimodal Examination of Atrial Fibrillation Substrate. JACC Clin. Electrophysiol..

[18] Roney C.H., Sillett C., Whitaker J., Lemus J.A.S., Sim I., Kotadia I., O’Neill M., Williams S.E., Niederer S.A. (2021). Applications of multimodality imaging for left atrial catheter ablation. Eur. Heart J. - Cardiovasc. Imaging..

[19] Si D., Wu Y., Xiao J., Qin X., Guo R., Liu B., Ning Z., Yin J., Gao P., Liu Y., Yang D., Cheng K., Chen T., Cheng Z., Lin X., Fang Q., Herzka D.A., Ding H. (2023). Three-dimensional High-Resolution Dark-Blood Late Gadolinium Enhancement Imaging for Improved Atrial Scar Evaluation. Radiology.

[20] Falasconi G., Penela D., Soto-Iglesias D., Francia P., Teres C., Saglietto A., Jauregui B., Viveros D., Bellido A., Alderete J., Meca-Santamaria J., Franco P., Gaspardone C., San Antonio R., Huguet M., Cámara Ó., Ortiz-Pérez J.-T., Martí-Almor J., Berruezo A. (2023). Personalized pulmonary vein antrum isolation guided by left atrial wall thickness for persistent atrial fibrillation. Europace.

[21] Dong J., Calkins H., Solomon S.B., Lai S., Dalal D., Lardo A.C., Brem E., Preiss A., Berger R.D., Halperin H., Dickfeld T. (2006). Integrated electroanatomic mapping with three-dimensional computed tomographic images for real-time guided ablations. Circulation.

[22] Richmond L., Rajappan K., Voth E., Rangavajhala V., Earley M.J., Thomas G., Harris S., Sporton S.C., Schilling R.J. (2008). Validation of computed tomography image integration into the EnSite NavX mapping system to perform catheter ablation of atrial fibrillation. J. Cardiovasc. Electrophysiol..

[23] Finlay M.C., Hunter R.J., Baker V., Richmond L., Goromonzi F., Thomas G., Rajappan K., Duncan E., Tayebjee M., Dhinoja M., Sporton S., Earley M.J., Schilling R.J. (2012). A randomised comparison of Cartomerge vs. NavX fusion in the catheter ablation of atrial fibrillation: The CAVERN Trial. J. Interv. Card. Electrophysiol..

[24] Nollo G., Cristoforetti A., Faes L., Centonze M., Del Greco M., Antolini R., Ravelli F. (2004). Comput. Cardiol. 2004.

[25] Cristoforetti A., Masè M., Faes L., Centonze M., Greco M.D., Antolini R., Nollo G., Ravelli F. (2007). A stochastic approach for automatic registration and fusion of left atrial electroanatomic maps with 3D CT anatomical images. Phys. Med. Biol..

[26] Tavard F., Simon A., Leclercq C., Pavin D., Hernandez A., Garreau M. (2009). 2009 16th IEEE Int. Conf. Image Process. ICIP.

[27] Shu L., Wang J., Long D., Lin C. (2017). An automatic and accurate registration method for electro-anatomical map and CT surface. Int. J. Med. Robot..

[28] Roney C.H., Pashaei A., Meo M., Dubois R., Boyle P.M., Trayanova N.A., Cochet H., Niederer S.A., Vigmond E.J. (2019). Universal atrial coordinates applied to visualisation, registration and construction of patient specific meshes. Med. Image Anal..

