# Supplementary material for: Multi-modal characterization of the left atrium by a fully automated integration of pre-procedural cardiac imaging and electro-anatomical mapping
Source: Int J Cardiol Heart Vasc. 2023 Oct 11;49:101276. doi: 10.1016/j.ijcha.2023.101276 (PMC10579959; doi:10.1016/j.ijcha.2023.101276)
Supplement: Supplementary data 1 [file mmc1.docx]

**Supplementary Materials**

**Supplementary Table S1**

|  |  | Median |  | 5^th^ – 95^th^ percentiles |
| --- | --- | --- | --- | --- |
| Patient 1 | Intraobserver variability | 0.95 |  | [0.24 – 2.84] |
|  | Interobserver 1 | 1.10 |  | [0.32 – 6.07] |
|  | Interobserver 2 | 1.07 |  | [0.32 – 6.25] |
|  |  |  |  |  |
| Patient 2 | Intraobserver variability | 1.00 |  | [0.26 – 7.89] |
|  | Interobserver 1 | 1.13 |  | [0.37 – 9.60] |
|  | Interobserver 2 | 1.17 |  | [0.33 – 7.06] |
|  |  |  |  |  |
| Patient 3 | Intraobserver variability | 0.99 |  | [0.18 - 3.52] |
|  | Interobserver 1 | 1.77 |  | [0.38 – 11.72] |
|  | Interobserver 2 | 1.59 |  | [0.37 – 11.85] |

Data represented in millimeters. Intraobserver variability shows the distance between two segmentations of the first observer (GPB). Interobserver 1 and 2 show the distances between the two segmentations by the first observer with respect to the second observer (SMC).
